# Has COVID-19 suppressed dengue transmission in Nepal?

**DOI:** 10.1017/S0950268822001790

**Published:** 2021-11-18

**Authors:** Basu Dev Pandey, Mya Myat Ngwe Tun, Kishor Pandey, Shyam Prakash Dumre, Pramananda Bhandari, Uttam Raj Pyakurel, Nayanum Pokhrel, Meghanath Dhimal, Pardip Gyanwali, Richard Culleton, Yuki Takamatsu, Anthony Costello, Kouichi Morita

**Affiliations:** 1 Everest International Clinic and Research Center, Kathmandu, Nepal; 2 Department of Molecular Epidemiology, Institute of Tropical Medicine, Nagasaki University, Nagasaki, Japan; 3 Department of Virology, Institute of Tropical Medicine, Nagasaki University, Nagasaki, Japan; 4 Central Department of Zoology, Tribhuvan University, Kirtipur, Nepal; 5 Central Department of Microbiology, Tribhuvan University, Kirtipur, Nepal; 6 Sukra Raj Tropical and Infectious Disease Hospital, Kathmandu, Nepal; 7 Epidemiology and Disease Control Division, Department of Health Services, Ministry of Health and Population, Kathmandu, Nepal; 8 Nepal Health Research Council, Kathmandu, Nepal; 9 Division of Molecular Parasitology, Proteo-Science Center, Ehime University, Matsuyama 790-8577, Japan; 10 Institute for Global Health, University College London, London, UK

**Keywords:** COVID-19, Dengue, Nepal

## Abstract

Following the report of the first COVID-19 case in Nepal on 23 January 2020, three major waves were documented between 2020 and 2021. By the end of July 2022, 986 596 cases of confirmed COVID-19 and 11 967 deaths had been reported and 70.5% of the population had received at least two doses of a COVID-19 vaccine. Prior to the pandemic, a large dengue virus (DENV) epidemic affected 68 out of 77 districts, with 17 932 cases and six deaths recorded in 2019. In contrast, the country's Epidemiology and Disease Control Division reported 530 and 540 dengue cases in the pandemic period (2020 and 2021), respectively. Furthermore, Kathmandu reported just 63 dengue cases during 2020 and 2021, significantly lower than the 1463 cases reported in 2019. Serological assay showed 3.2% positivity rates for anti-dengue immunoglobulin M antibodies during the pandemic period, contrasting with 26.9–40% prior to it. Real-time polymerase chain reaction for DENV showed a 0.5% positive rate during the COVID-19 pandemic which is far lower than the 57.0% recorded in 2019. Continuing analyses of dengue incidence and further strengthening of surveillance and collaboration at the regional and international levels are required to fully understand whether the reduction in dengue incidence/transmission were caused by movement restrictions during the COVID-19 pandemic.

## Background

Nepal, home to 30 million people, shares borders with India and China. The first case of COVID-19 in the country was reported on 23 January 2020, and was followed by a first wave of the disease that peaked in October 2020 [1]. Following a surge of cases in India, the Delta variant entered Nepal in April 2021 leading to the highest numbers of cases and deaths. This was followed by a third wave caused by the Omicron variant in December 2021 [1]. Hospitals became crowded and almost all hospital beds in Kathmandu were occupied by COVID-19 patients with the highest number of daily deaths (246) on 19 May 2021 [1]. Many patients were turned away due to a lack of oxygen, intensive care unit beds and ventilators. The government of Nepal declared a health emergency and imposed a nationwide lockdown that started on 23 May 2021, and which lasted for 6 months during 2021 [1]. Following the second wave, the long open border between India and Nepal, lacking strict health regulations and insufficiently monitored adherence to public health advice may have contributed to a large third wave of infections. The introduction of COVID-19 vaccination and the strengthening of the health system perhaps contributed to the better management of the third wave in Nepal, which resulted in fewer hospitalisations and deaths than the previous waves. By the end of July 2022, a cumulative 986 596 cases had been confirmed by real-time polymerase chain reaction (RT-PCR) with 11 967 deaths resulting in a case fatality rate of 1.2% [2]. Over 70% of the total population had received at least two doses of the COVID-19 vaccine by July 2022 [2].

The COVID-19 pandemic has had an unprecedented impact on public health systems worldwide, and has indirectly affected the epidemiology of other infectious diseases, including dengue. Apart from COVID-19, dengue is the most common outbreak-prone arboviral disease in the tropical and subtropical regions of the world. Despite the fact that there has been an increase in its transmission intensity, and that its range is increasing, dengue has never been declared a public health emergency of international concern by the WHO [3].

Dengue, a mosquito-borne neglected tropical disease, caused by a mosquito-borne virus is particularly prominent in South-East Asia, South Asia and Latin America where cases, deaths and disability-adjusted life years (DALYs) are increasing [4, 5]. Urbanisation, climate change, water scarcity and increased human mobility may impact on the global burden of dengue [5]. Dengue has been endemic in Nepal since it was first reported in the Chitwan district in 2004 [6]. As in South-East Asian countries, it is a major public health problem due to its logarithmic escalation over the past decade [4, 5].

The first dengue outbreak in Nepal occurred in 2006 across nine districts and involved 32 cases. The presence of the vector *Aedes aegypti*, was confirmed in these districts [7–9]. The first-*ever large-scale* outbreak of dengue occurred in 2010, and involved the dengue virus (DENV)-1 and -2 serotypes. This outbreak was followed by cyclical epidemics every 2–3 years with the largest outbreak observed in 2019 [7]. Dengue has been endemic in the lowland Terai region for several years and it is currently emerging and expanding into the upland regions [9], where dengue vectors have been reported up to 2000 m above sea level [10].

The number of cases of dengue in Nepal usually start increasing by June, this coinciding with the rainy season, and peak in the month of November before gradually declining to undetectable levels by December [7–9]. It is often difficult to differentiate between dengue and COVID-19 since some symptoms are similar and both infections may be found in the same regions. The dual burden of these diseases has the potential to result in a catastrophic situation in a resource-constrained country like Nepal [11]. At the time of the COVID-19 pandemic, the majority of human resources and logistics were diverted to fight this disease, resulting in the neglect of measures against dengue [3, 11].

## Current dengue situation in Nepal

Dengue cases have been reported every year with seasonal outbreaks occurring in 2006, 2010, 2013, 2016 and 2017 involving 32, 917, 683, 1527 and 2111 cases, respectively [7–9]. The disease is now endemic in most parts of the country and all four serotypes of the DENV have been observed since 2006 [7]. DENV-1 was the prominent serotype between 2010 and 2016, while the outbreaks in 2013, 2017 and 2019 were solely by DENV-2 [7–9]. In recent years, Nepal has witnessed an upsurge of vector-borne diseases, mainly dengue and chikungunya, of increasing severity and with expansion towards hilly regions [9]. In 2019 a large dengue epidemic started in eastern Nepal and moved quickly to populous Kathmandu, with cases occupying the majority of hospital beds [8, 9]. It affected 68 out of 77 districts, with 17 932 cases and six deaths reported, although many more cases and deaths were probably missed [7–9]. The healthcare system almost collapsed, causing widespread public panic and fuelling demands for better diagnosis and management.

Nepal faced the additional public health and socio-economic impact of the COVID-19 pandemic immediately after the dengue epidemic in 2019. The densely populated Kathmandu valley was expected to have a much bigger dengue epidemic in the coming years due to unplanned urbanisation, poor water management, a favourable climate for mosquito breeding and weak health system infrastructure to respond [12].

## Is there a threat of a bigger dengue epidemic in Kathmandu?

Kathmandu is inhabited by 3 million people and is located at a mean elevation of about 1300 m above sea level. This subtropical valley has a temperature of 19–27 °C in summer and 2–20 °C in winter with a maximum temperature of around 35 °C in summer and a minimum of around −3 °C in winter and 75% average annual humidity [13]. However, a recent estimate suggests that the average annual maximum temperature of Nepal has risen by 0.056 °C over the past 40 years, with greater warming at higher altitudes [9, 13]. The average rainfall is 1400 mm between June and August.

Kathmandu is the largest municipality with a population density of 13 225 people per square kilometre with rapid population growth. It is ethnically and culturally diverse, has the only international airport and is the country's biggest business hub. Many cities within the region have become ideal breeding sites for *Aedes* mosquitoes. Inadequate health infrastructure, trade and transit from dengue-infested areas, relentless and unplanned development and climate change and rising temperatures in mountain areas all contribute to the spread of dengue in urban areas [9, 12]. There is a high risk of a rapid upsurge of dengue cases due to *Aedes* mosquitos which have the potential to rapidly spread infection in a closely packed population like Kathmandu.

**Fig. 1.**
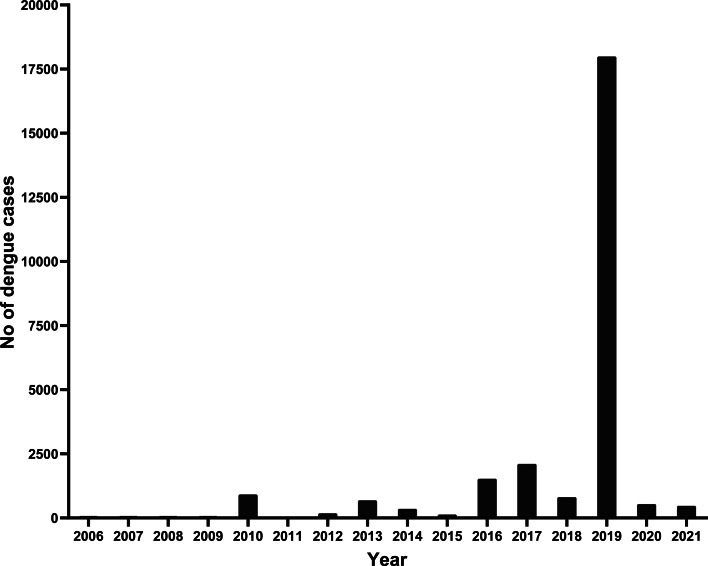
Number of dengue cases in 2006–2021 in Nepal: data obtained from Epidemiology and Disease Control Division, Department of Health Services, Ministry of Health and Population, Nepal.

A resurgence of vector-borne diseases was thought to be a real threat during the COVID-19 pandemic because public health staff and resources for vector control programmes were diverted to the pandemic response. Health planners expected a much bigger epidemic of dengue in Kathmandu [12]. Surprisingly, there were few cases of dengue reported to the Epidemiology and Disease Control Division (EDCD), Department of Health Services, and Ministry of Health and Population during the COVID-19 pandemic in 2020 and 2021 (530 and 540 cases, respectively) (Fig. 1). Kathmandu reported just 11 and 52 cases in 2020 and 2021, far lower than the 1463 recorded in 2019. The 15–49 age group was affected mostly and there was no severe dengue or death reported to EDCD during the pandemic period of 2020–2021. To verify EDCD data normally received from the Early Warning and Reporting System (EWARS), a total of 374 serum samples collected from three ecological regions during the pandemic and in the COVID-19 vaccinated individuals of the dengue endemic region were investigated in 2020 and 2021. This showed the positive rate of immunoglobulin M (IgM) antibodies to be 3.2% during the pandemic period (2020 and 2021). However, during the pre-pandemic era, the IgM enzyme-linked immunosorbent assay (ELISA) positivity rate was 26.9% in 2017 and 40% in 2019 [9, 14, 15]. Out of the 374 samples analysed, 27 (7.21%) samples were IgG ELISA positive. One (0.5%) of the 199 serum samples tested positive for DENVs by using RT-PCR, which is far less than the 111 (57.0%) out of 195 samples that tested positive in 2019 [15].

## Discussion

A substantial decline in dengue incidence was observed in Nepal during the COVID-19 pandemic years 2020 and 2021. The imposition of strict restrictions and changes in human movement during the pandemic were probably the most important factors in decreasing the number of cases. School closures and declines in international tourists and non-residential trips, with limited social mixing, are associated with reduced dengue risk in South-East Asian and Latin American countries [16]. The overall dengue incidence in Nepal was rapidly increasing since its introduction in the country until the COVID-19 pandemic occurred. This trend was very similar to what has been reported in several South Asian countries where high-middle and high socio-demographic index regions have seen age-standardised incidence, deaths and DALYs rates accelerated [3, 4]. Geo-sentinel surveillance over the past two decades has documented increasing trends of arboviral diseases in South Asia most frequently DENV infection in travellers with increasing morbidity to an average of 159 cases per 1000 travellers during the epidemic [17]. Diagnosis remains difficult as febrile illnesses with common presentation are increasing globally [18].

There was a 46% increase in dengue cases from 2015 to 2019, with a prominent peak in 2019 from Association of Southeast Asian Nations (ASEAN) region [19]. More recent studies during the pre-COVID-19 period (2015–2019) and COVID-19 period (2020–2021) showed average dengue incidences across the 22 major dengue-endemic Asian countries including Nepal and Latin American countries decreasing by 16% during the pandemic period compared to the pre-COVID-19 time (2015–2019), although sporadic increases in the incidences were observed in some countries including Singapore [20]. A few cases of COVID-19 and dengue co-infection have been reported from Nepal and other dengue-endemic countries [11, 19].

It seems likely that one of the biggest drivers of the decline in dengue during the COVID-19 pandemic were the lockdowns imposed on the population (Nepal imposed 5- and 6-month lockdowns in 2020 and 2021), which would have resulted in vastly reduced entomological inoculation rates, and the cessation of the spread of the virus through human movement between regions. It is also possible that during the pandemic, people maintained a cleaner environment resulting in less opportunity for the breeding of mosquitoes. Another factor may be that attention to detect, test and trace for COVID-19 may have led to significant under-reporting of dengue.

Understanding the impact of lockdown factors on transmission and the apparent decline of dengue incidence is important for the post-pandemic period. Understanding exactly how lockdowns lead to decreases in dengue transmission is of paramount importance when designing the post-COVID-19 pandemic dengue control programme [21]. With restrictions now over, and mobility and social mixing resumed, dengue will certainly become dominant again and could spread to the upland regions. Efforts to detect and prevent cases through strengthening the surveillance system for vector-borne diseases are crucial for the sustained reduction of dengue in Nepal.

Nepal practices a EWARS under the EDCD which regularly receives information on outbreak-prone vector-borne diseases from 118 sentinel sites throughout the country. Amidst the burden of the COVID-19 pandemic, densely populated cities face the further challenge of widespread endemic diseases such as dengue resurging following the cessation of restrictions and the resumption of regular international flights. The government needs to urgently strengthen effective measures to combat dengue fever. EWARS need to be further strengthened and expanded for efficient surveillance of dengue and other outbreak-prone vector-borne diseases. Dengue surveillance should be integrated into the nationwide health management information system, with an arrangement of key indicators monitored at all levels in the three levels of government healthcare. Health officials, and particularly vector control officers should identify potential vector breeding sites and work on interventions to drain them as early as possible. Community engagement for search and destroy activities aimed at eliminating superfluous water containers in which *Aedes* mosquitoes may breed should also be instigated.

Despite the fact that Nepal is a popular tourist destination, there is no systematic tracking and recording of imported dengue cases. Interestingly, the major port of entry for international tourists is the capital city of Kathmandu was considered to be ‘dengue-free’ by tourists. However, in recent years, autochthonous transmission of dengue is common in Kathmandu. Therefore, Kathmandu should be considered a dengue risk area and travellers to Nepal should be aware of the potential threats of infection during the rainy/post-rainy season [4, 22].

## Recommendation

The emergency action plan needs to be revised for outbreak preparedness at the central, provincial and municipal levels. Municipalities should prioritise education of female community health volunteers and other local volunteers on vector-borne diseases including dengue and on the appropriate response to their presence. Mass communication is essential to educate the public on how to protect themselves from mosquito bites using strategies, such as mosquito nets, insect repellents and appropriate clothing, as well as actively intervening to control vector populations by ensuring proper waste disposal and regular maintenance of public places (a major issue in Kathmandu Metropolitan City).

In the public and private sectors across the nation, more than 100 molecular testing facilities have been set-up to test for COVID-19, and these could be utilised for molecular surveillance of dengue, including the identification of serotypes. The government should provide the most sensitive and specific rapid diagnostic test for dengue and antigen tests for COVID-19 in hospitals. Hospitals should increase their accessibility for dengue patients by creating more space and resources for prompt treatment. Health workers should be provided training for the appropriate management of cases.

## Conclusion

Enhancing case detection capacity for dengue and COVID-19 and the strengthening and maintenance of disease control systems are of utmost importance during the pandemic. There is an urgent need to understand why the COVID-19 epidemic in Nepal has led to drastic reductions in dengue cases, and to apply this knowledge to future dengue control programmes.

## Data Availability

The data used in the current study are available from the corresponding author upon reasonable request.
